# Supplementary material for: Patterns of Intron Gain and Loss in Fungi
Source: PLoS Biol. 2004 Nov 30;2(12):e422. doi: 10.1371/journal.pbio.0020422 (PMC532390; doi:10.1371/journal.pbio.0020422)
Supplement: Table S1 — Also available at http://genes.mit.edu/NielsenEtAl/. (4.3 MB ZIP). [file pbio.0020422.st001.zip › NielsenEtAl/html/925.html]

AN5354.1.NCU06080.1.MG04975.1.FG09186.1


```
 CLUSTAL W (1.82) Multiple Sequence Alignments - Introns Inserted


Sequence 1: AN5354.1	329 aa
Sequence 2: MG04975.1	418 aa
Sequence 3: NCU06080.1	347 aa
Sequence 4: FG09186.1	334 aa
Alignment Length: 459 aa
Number Identitical Residues: 100 aa
Alignment Score (without introns) 5887


MG04975.1 	-------------------------MMKEAI~VTRGP~EVTIADSEIPSPGPDQLVIRVV
NCU06080.1	--------------------------MKEAI0IIDSP~---IP-----SPNDDQVLIRVI
FG09186.1 	MLYRLSNRTALYRLQALSRQVRNIGMMKEAI~VSSGP~EVEIVDSPIPKAGPGQVVTKIA
AN5354.1  	--------------------------MKEAI~VPKGL2LITIW------PDPVDYEYNHN
          	                          ***** :  .     *       ..  :   .  

MG04975.1 	VSGSNPKDW1KVTEWLG--KTMNTGDDIAGIVHSVGSNVWEFKPGDRVASFHEMLTPGGS
NCU06080.1	VSGCNPKDW~KLPGMMPDKPPLNQGDDIAGIVEKVGSRVWEFKPGDRVAAFHEMLTPHGS
FG09186.1 	FAASNPKDW2KRPLYWGAKGTGNQGDEHAGVVHEVGEGVYEFNPGDRVAAMHEMKTPGGS
AN5354.1  	NSDEKVSDR~KLPEWLD--QAGNQGDDIAGIVEAVGDNVTEFKPGDRVMAFHEMTKPGGS
          	 :  : .*  * .       . * **: **:*. **. * **:***** ::*** .* **

MG04975.1 	FAEYALGRQHTTFHLPSKVSFEE1AATLPLAYMTSALGLHLRLGLPVPWTPATEP-----
NCU06080.1	WAEYAVAWQHTTFHIPKETSFEE~AAALPLAAMTAAVGLFSRLGLPEPWVKAVSDEERQN
FG09186.1 	YAEYGLSPAYTTFKLPDNTSFQE1GAAIPLTALTAACALYARLKLPEPWLPVPDSEK---
AN5354.1  	YAEYALAWQHTTAHLPKHISFEE1AATIPLAALTSVSALYHHLQLPTPWSPAASS-----
          	:***.:.  :** ::*.. **:* .*::**: :*:. .*. :* ** **  . .      

MG04975.1 	TPLVVYGGASAVGAFVIKLAVKSNIHPIITVAGRG-ADFVEGLIDRSKGDTIVDYRKGDD
NCU06080.1	TPLVIYGASSAVGYYALQLAKRSNIHPLICIAGRAGGERIAPLLDQDKGDVLIDYREGEQ
FG09186.1 	IPLVIWGASSAVGSYAIQLAKRSNIHPLICIAGKA-QEHVESLIDRTKGDTVIDYRKGRN
AN5354.1  	IPLVIYGGASAIGAFAIQLARKSNIHPIIAIAGRS-APFVEGLIDRSKGDTIIDYRQGDE
          	 ***::*.:**:* :.::** :*****:* :**:.    :  *:*: ***.::***:* :

MG04975.1 	AVVQGIRDALKGAELKYAYDATSEHNSYFNIAKAMST----GGK---ITTVLPAKD-EAL
NCU06080.1	AVVEGIKKALNGRPLLHALDAVSEKGTPEHLAQVLSQPVKKGDKEVRAKATFVLFGKMGV
FG09186.1 	TVIQEMKHHLGNQKLEYAFDAISEGGSYQTICDVLDK-----------------------
AN5354.1  	AVISGIKDALNGAELFHTYDCVSEGSSFVNLGKVVAP----GGK---IVTVLPFQDYPGV
          	:*:. ::. * .  * :: *. ** .:   : ..:      ...     :     .  . 

MG04975.1 	PDNVEITTTMVSTAHGDESD~FAYAWFRLLARWLDEGTFKPHPHEVVEGGLNGVEKGLKG
NCU06080.1	PDFVEQSTTLVGSVHQDEKD~FGYVWYRYMARGLQEGWFKAQRTEVLPGGLAGVQEGLER
FG09186.1 	-----------------TTD1FGYVFTRYFSKGLEDGWLKAHPQEVIPGGLEGIQQGLEN
AN5354.1  	PEHVQVIKNSVGCVHQDQKD~LGYVYFRYLAKGLQDGWFKPHPHQVVPGGLGGLETALCN
          	..  .  ..  .    . .* :.*.: * ::: *::* :*.:  :*: *** *:: .*  

MG04975.1 	LKEGKASAVK2LEGCKRLEPSHAYLRQKVPSSMQMSEVGIAPIWSRWAALAMLSSYRCAV
NCU06080.1	LKDGKASAVK~YV-----------YRIQETEGVERSEE----------------------
FG09186.1 	LENGKASAVK~YV-----------YKIVDTPGVVP-------------------------
AN5354.1  	LRDGKASATK~YV-----------VRIGETSGL---------------------------
          	*.:*****.*               :   . .:                           

MG04975.1 	FRLFTPSPPRRSHRGPAAKSRYGGLVGSGDWLSMVECYQSTPSSL
NCU06080.1	---------------------------------------------
FG09186.1 	---------------------------------------------
AN5354.1  	---------------------------------------------
          	
```
